# A Lofty Imagination

**Published:** 1888-05

**Authors:** 


### A LOFTY IMAGINATION.

A reporter of the San Francisco *Chronicle* on one occasion asked Dr. Swan, "Do you believe in the Mind-cure?" He replied: "Well, the mind-cure is adopted very often by the medical profession, and it is a very valuable aid to doctors sometimes. I had a patient once, a lady of nervous temperament, who had for a long time suffered from restlessness, nervousness, sleeplessness, and other causelessnesses. There was nothing really the matter with her; her trouble was in the imagination. I could not cure her at all. At last one evening I said to her: 'Now I never like to give morphine or any kind of opium. It is excessively dangerous, and only as a last recourse do I administer it. I have decided to administer it to you, I am a little nervous about the result, and you must be very careful in using it.' And I went to the faucet and drew a glass of water and compounded with great care and seriousness a slightly-colored mixture of which I had brought the materials. 'Here, take this teaspoonful,' I said. 'Now, if you don't get to sleep in half an hour, take another teaspoonful, if that does not work, wait an hour and try another, but don't take any more for two hours, because this is cumulative, and there's enough in this to kill the family. Please be very, very careful,' and I left her. Next day I called.

"'O doctor,' she said, 'I am so much better. The first teaspoonful did no good; so I took another, and that worked like a charm. I slept beautifully and got up feeling infinitely better.'

"'I am glad,' I said, 'you've had enough. I will throw the rest away for it is excessively dangerous. It was after a couple of years of good health I confessed to her that all in the world she had taken was a teapsoonful of brown sugar and water. She was so mad she almost fell sick again"

